# Non-contiguous Rare Presentation of Spinal Tuberculosis: A Case Report

**DOI:** 10.7759/cureus.44881

**Published:** 2023-09-08

**Authors:** Abdullah S Binsaeedu, Nehal V Sadi, Sagal Yusuf, Salma Yusuf, Humayun Youshay

**Affiliations:** 1 Internal Medicine, Alfaisal University College of Medicine, Riyadh, SAU; 2 Internal Medicine, Saint James School of Medicine, Chicago, USA; 3 Internal Medicine, Windsor University School of Medicine, Illinois, USA; 4 Nephrology, Swedish Hospital, Chicago, USA

**Keywords:** case report, non-contiguous multilevel spinal tb, extra-pulmonary tuberculosis, pott's disease, spinal tuberculosis

## Abstract

Spinal tuberculosis (TB), also known as Pott's disease, is a severe form of extrapulmonary TB that affects the vertebral bodies and intervertebral discs. While the typical presentation involves the contiguous involvement of multiple vertebrae, atypical forms, such as non-contiguous multilevel spinal TB (NMLST), can occur. However, diagnosing spinal TB poses challenges due to its gradual onset, nonspecific symptoms, and varying imaging results. The timely diagnosis and treatment of spinal TB are critical to prevent serious consequences, including vertebral damage, irreversible neurological impairment, or even death. In this report, we present the case of a 58-year-old South Asian female who presented with several months of back pain, fatigue, and weight loss. Despite initially negative TB test results, spinal magnetic resonance imaging (MRI) raised suspicion of NMLST, which was later confirmed by bone biopsy. This case highlights the complexities of diagnosing and managing atypical spinal TB presentations while discussing the case findings and reviewing relevant research.

## Introduction

Spinal tuberculosis (TB), also known as Pott's disease or tuberculous spondylitis, is a rare but serious form of extrapulmonary TB that affects vertebral bodies and intervertebral discs [1]. It is caused by the hematogenous or lymphatic spread of Mycobacterium tuberculosis (MTB) from a primary infection site, usually in the lungs or urogenital tract [2]. While pulmonary TB is the most common form of TB, approximately 16% of TB cases are extrapulmonary, with 10% involving musculoskeletal structures [3,4]. Spinal TB accounts for nearly 1% of all TB cases, and it is the most common and dangerous form of musculoskeletal TB, affecting the thoracolumbar region, followed by the cervical and sacral regions [5]. Spinal TB can lead to vertebral collapse, spinal deformity, paraspinal abscesses, and neurological complications due to cord compression [6]. Constitutional symptoms, such as fever and weight loss, have also been reported in the literature [7]. The typical pattern of spinal TB is the contiguous involvement of two or more vertebrae with the destruction of the intervening disc [6]. However, atypical forms of spinal TB can occur, such as non-contiguous multilevel spinal TB (NMLST), with skipped lesions and preserved discs. NMLST is rare and can mimic other conditions such as pyogenic spondylitis and malignancy [8]. Our case report discusses a middle-aged female patient from Southeast Asia with NMLST. We also reviewed relevant literature and highlighted the importance of considering spinal TB in the differential diagnosis of patients with chronic back pain and multiple vertebral lesions.

## Case presentation

**Figure 1:**
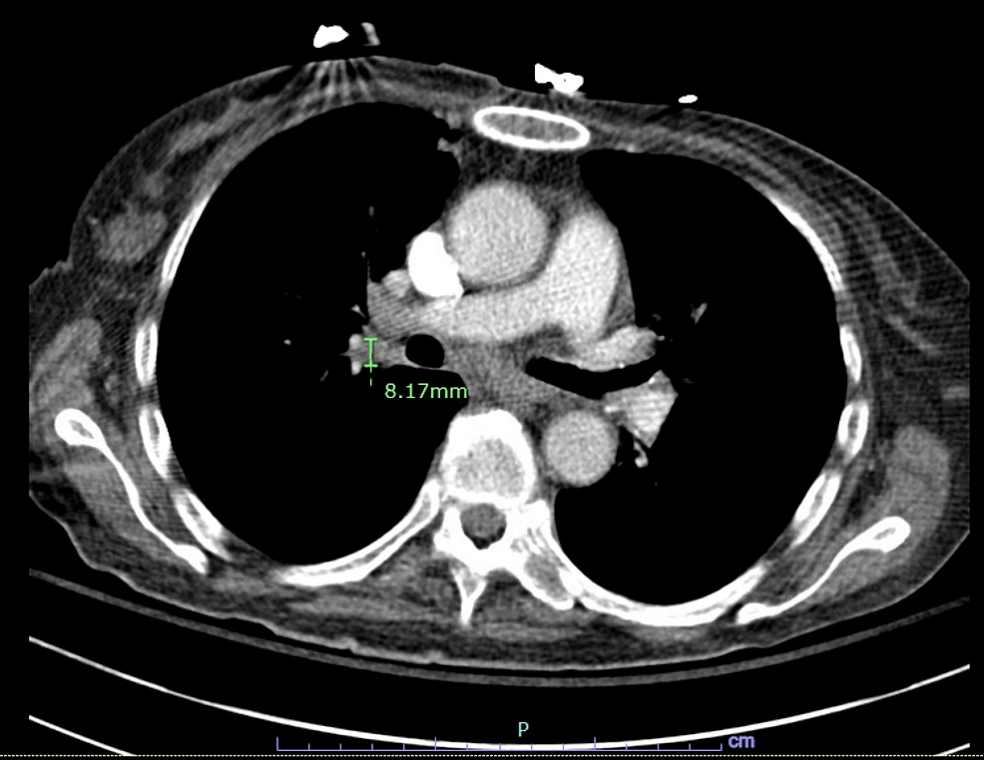
Computed tomography (CT) scan of the chest with contrast showing hilar adenopathy (green measurement)

A 58-year-old South Asian female presented to the emergency department complaining of nine months of failure to thrive, poor appetite, fatigue, weakness, back pain, intermittent productive cough, and anemia. The patient had lost approximately 60 pounds (27.2 kg) within the past year and had experienced multiple falls. Her medical history included type 2 diabetes mellitus and hypothyroidism. She had traveled to India a year prior to the onset of her symptoms. Initially, workup to identify the source of anemia included a colonoscopy, esophagogastroduodenoscopy, chest X-ray, and TB test, which were unremarkable. Inflammatory markers were elevated, including erythrocyte sedimentation rate (30 mm/h) and C-reactive protein levels (14 mg/L). Human immunodeficiency virus (HIV) test yielded a negative result. The computed tomography (CT) scan of the chest with contrast revealed hilar adenopathy (Figure 1).

**Figure 2:**
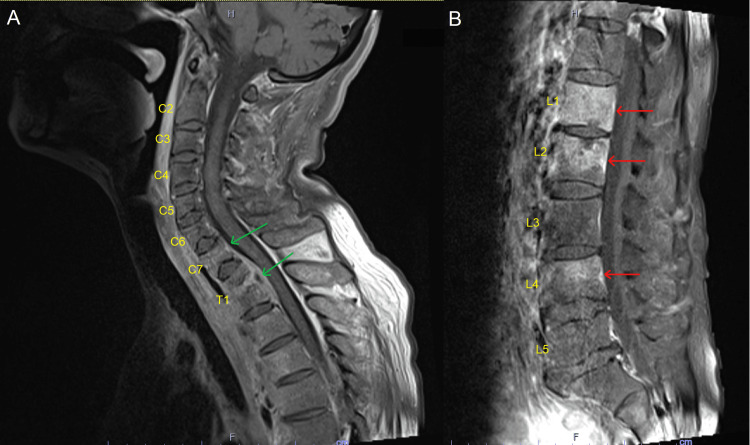
T1-weighted magnetic resonance imaging (MRI) of the spine (A) A stable destructive process at the C7-T1 vertebral levels (green arrows). (B) A stable destructive process at the L1-L4 vertebral levels (red arrows).

Additionally, spinal magnetic resonance imaging (MRI) exhibited multifocal abnormal marrow enhancement involving the C7-T1 and L1-L4 vertebral levels corresponding to metastatic disease upon initial assessment (Figure 2). An underlying infectious etiology was suspected although definitive cultures were not obtained. CT-guided bone biopsy with deoxyribonucleic acid (DNA) amplification test confirmed TB. The patient was started on rifampin, isoniazid, pyrazinamide, and ethambutol for spinal TB and continued amoxicillin-clavulanic acid for bilateral lung infiltration.

## Discussion

Atypical forms of spinal TB can show the involvement of posterior elements (pedicle, transverse and spinous processes, and lamina), destruction of vertebral bone without intervertebral disc involvement, noncontiguous skip lesions affecting multiple spinal regions, and extradural lesions with preserved vertebral bone [8]. NMLST found in our case is a rare and atypical form of spinal TB that may have a higher incidence than previously reported [9], especially with novel imaging approaches such as whole-spine MRI. The incidence of NMLST ranges from 1.1% to 71.4% [9]. The discrepancy in the incidence rate may be due to the different imaging modalities used. This case report presents a patient with NMLST involving three segregated levels of the vertebral body and posterior elements in the cervical, thoracic, and lumbar regions with paravertebral and epidural abscesses and intact intervertebral discs. Spinal TB diagnosis is often delayed, with an average of one year and seven months from presentation to diagnosis [10]. Symptoms progress slowly, lasting from two weeks to several years before diagnosis. It occurs mainly in males with a mean age of 43.4 years [10]. Symptoms vary, depending on disease stage and location, but most commonly include back pain, fever, weight loss, neurological abnormalities, and night sweats [10]. The thoracic spine is the most frequently affected region [10]. Immunocompromised individuals, such as diabetics and patients taking steroids, as well as individuals residing in low socioeconomic areas with endemic TB, are often at a higher risk of TB [11]. Complications can result in neurologic deficits and paraplegia, with an incidence rate ranging from 23% to 76% [12]. Early diagnosis of spinal TB is critical for preventing irreversible neurological damage or death.

Diagnosis can be challenging due to the overlap of symptoms with other conditions and the potential for obtaining negative results on initial testing. However, a positive TB history, an elevated erythrocyte sedimentation rate, and the utilization of more advanced tests, such as a DNA amplification test, as demonstrated in our case, can significantly contribute to the diagnostic process [13]. DNA amplification testing is a fast and accurate diagnostic tool for TB [13]. Radiographic changes can also aid in diagnosis, with MRI being the preferred modality because it may detect the characteristic features of spinal TB. These distinctive features include spinal lesions originating from the vertebral endplate, subligamentous spread, multiple vertebral body involvement, and paraspinal abscess formation [14]. Plain radiography may not show early changes, as seen in our case, until at least 30% bone mineral loss [10]. In contrast, CT is useful for defining soft tissue abscesses but less accurate for identifying epidural extension and neural involvement compared to MRI [10].

Spinal TB has a favorable prognosis if treated early, with a cure rate of 90% [7]. In our case, the initiation of antibiotic therapy was delayed until confirmation of TB infection based on the pathology results due to an uncertain diagnosis. Anti-TB agents are the cornerstone of spinal TB treatment, with combination therapy of four drugs: isoniazid, rifampicin, pyrazinamide, and ethambutol. All four are administered for the first two months, followed by isoniazid and rifampicin for the subsequent 10 months, with a total treatment duration of 12 months [7]. Surgery is recommended only when necessary, such as in cases with neurological issues, spinal instability, significant kyphosis, refractory pain, or medical treatment failure. However, the recommended treatment for NMLST must be clarified, and the MTB susceptibility pattern must be identified to guide treatment. Furthermore, there is a need for evidence-based guidance regarding treatment protocols, particularly for surgical procedures for NMLST.

## Conclusions

In conclusion, this case underscores the significance of including spinal TB in the list of differential diagnoses for patients presenting with chronic back pain and multiple vertebral lesions, particularly in atypical presentations. It also emphasizes the necessity of adopting a multidisciplinary approach to diagnose and manage spinal TB, involving infectious disease specialists, interventional radiologists, and orthopedic surgeons, as such collaboration is imperative for optimizing patient outcomes.
